# Effect of Interval Time after Subintimal Plaque Modification on the Success Rate of Future Recanalization for Chronic Total Occlusion Percutaneous Coronary Interventions

**DOI:** 10.31083/RCM26991

**Published:** 2025-04-18

**Authors:** Ze Zheng, Xiaowen Bo, Songyuan He, Hongyu Peng, Ping Wang, Shujuan Cheng, Qian Fan, Jinghua Liu

**Affiliations:** ^1^Department of Cardiology, Beijing Anzhen Hospital, Capital Medical University, Beijing Institute of Heart, Lung, and Blood Vessel Diseases, 100069 Beijing, China

**Keywords:** chronic total occlusion, subintimal plaque modification, percutaneous coronary intervention

## Abstract

**Background:**

Chronic total occlusion (CTO) is a complex and difficult type of coronary lesion for which elective secondary intervention after subintimal plaque modification (SPM) can improve the success rate. This study sought to determine the most appropriate timing for secondary interval interventions to maximize the benefit to the patient.

**Methods:**

This study retrospectively included patients who failed their first CTO percutaneous coronary intervention (PCI) at Beijing Anzhen Hospital Department of Cardiology from January 2019 to December 2022. We reviewed the clinical characteristics, procedural features, and outcomes of patients who underwent SPM and returned to our institution for a second CTO-PCI.

**Results:**

Of the 2847 patients who visited our institution between January 2019 and December 2022, 528 underwent SPM and returned to our institution on an elective basis for a secondary procedure. Of these, 236 procedures were performed within 30 days (Group I), and 292 were performed between 30 and 90 days (Group II). After the intervention, the occluded segment was successfully opened in 170 (72.0%) Group I and 248 (84.9%) Group II participants. When analyzing the factors for operational failure, we found that different intervals, diabetes mellitus, hyperlipidemia, and a history of previous PCI or percutaneous coronary angioplasty (PTCA) were the reasons for the secondary intervention failure. When analyzing the safety of the procedure, we found that pericardial effusion was the most common complication after the procedure, with an incidence of 7.4%. There was no notable variation in the incidence of pericardial effusion between the two groups, 8.9% vs. 6.2% (*p* = 0.232).

**Conclusions:**

Higher success rates were observed when secondary procedures were performed between 30 and 90 days instead of within 30 days after the initial CTO-PCI SPM, with no significant difference in safety noted between the two groups.

## 1. Introduction

In previous years, coronary artery disease (CAD) was associated with increased morbidity and mortality [1, 2]. Nowadays, the number of deaths is gradually decreasing due to the timely opening of occluded or narrowed blood vessels, using percutaneous coronary intervention (PCI) and percutaneous coronary angioplasty (PTCA). However, there is a special lesion type of coronary lesion, chronic total occlusion (CTO), which is seen in 15–25% of CAD patients. The main manifestations of CTO are an occlusion with the absence of antegrade flow through the lesion with a presumed or documented duration of ≥3 months [3]. Given the challenges in accurately delineating the vascular cavity in CTO patients, the initial success rate of opening the occluded segment during procedures ranges from 70–90%, as is often accompanied by an increased incidence of complications [4, 5].

Therefore, CTO-PCI has failed or the potential risks may exceed the expected benefits, subintimal plaque modification (SPM) can be used as a supportive treatment strategy to modify the vascular anatomy with an appropriately sized balloon to plan subsequent interventions [6, 7, 8]. During subsequent interventions, SPM may result in either partial vascular healing or an increase in anterograde flow, which increases the success of subsequent PCIs [3, 6, 9].

At present, the research on SPM is still relatively limited, and the most optimal time to proceed with a second intervention is unknown. Appropriate timing of the second operation may lead to a higher surgical success rate for patients. This study aims to clarify the optimal time of the second operation after SPM by exploring the characteristics of CTO patients who undergo SPM at different times.

## 2. Methods

### 2.1 Study Populations

This retrospective study consecutively included patients who underwent SPM from January 2019 to December 2022 in the Department of Cardiology at Beijing Anzhen Hospital (Beijing, China) after a CTO-PCI procedure. Inclusion criteria were as follows: (1) The initial, subsequent CTO-PCI attempts and SPM during the procedures were undertaken by experienced cardiologists in Beijing Anzhen Hospital. (2) The repeat coronary angiography/intervention was taken within 90 days after SPM. Exclusion criteria were as follows: (1) patients aged <18 or >80 years old; (2) the presence of acute or chronic inflammatory diseases; (3) no dual anti-platelet therapy due to contraindications; (4) prior history of chronic renal insufficiency, malignancy, and a life expectancy of no more than 3 months.

### 2.2 Definitions and Study Endpoints

The primary endpoint was recanalization after the SPM procedure with thrombolysis in myocardial infarction (TIMI) grades 2–3 flow on angiography during an average of 48.5 days of follow-up. SPM was defined as a procedure performed which when the distal lumen re-entry fails or side branches cannot be recanalized, and balloon angioplasty is performed in the dissection planes to restore some antegrade flow, but no stenting is performed. Repeat angiography after 1.5–4 months revealed restoration of antegrade flow and healing of the dissection, allowing crossing of the lesion and successful recanalization [6]. CTO was defined as complete coronary occlusion of more than 3 months duration with TIMI flow grade 0 [10]. All the characteristics of coronary angiography including locations of the CTO, morphology of the stump, calcification at the site of the occlusion, vessel tortuosity and grade of collaterals, were evaluated by two experienced cardiologists. Calcification at the site of the occlusion, vessel tortuosity, retrograde procedure, antegrade dissection reentry (ADR), and calcification were defined according to the 2019 Consensus Document from the EuroCTO Club [10, 11].

Technical success was defined as successful CTO revascularization with achievement of <30% residual diameter stenosis within the treated segment and restoration of TIMI grade 3 antegrade flow [3]. Procedural complications were defined as donor vessel dissection, vessel perforation, pericardial tamponade, pericardial tamponade requiring pericardiocentesis, emergent PCI, and emergent coronary artery bypass grafting [12]. Major adverse cardiovascular event (MACE) was defined as in-hospital death, myocardial infarction, emergent cardiac surgery, stroke, or clinical perforation. Clinical perforation was defined as any perforation requiring treatment. Myocardial infarction (MI) was defined using the Fourth Universal Definition of Myocardial Infarction (type 4 MI).

### 2.3 Data Collection

Clinical characteristics of the study patients were collected including gender, age, medical history, medication history, smoking, laboratory examinations, transthoracic echocardiography, and coronary angiography findings. SPM characteristics including stingray balloon, balloon-to-vessel ratio, and the length of subintimal angioplasty were collected. The J-chronic total occlusion score (J-CTO) score was calculated according to the method of Morino *et al*. [10]. Detailed information about coronary angiography and procedures was evaluated by two experienced cardiologists. In addition, postoperative complications were recorded (i.e., death, coronary artery perforation, stroke, acute stent thrombosis, emergency surgery, and bleeding at the access site).

### 2.4 Statistical Analysis

Categorical variables were summarized as numbers (percentages) and compared using the chi-square test. Continuous data were presented as mean ± SD or median (interquartile ranges) and analyzed by the Student’s *t*-test or the Mann–Whitney U test. For continuous variables, normally distributed data were evaluated using the Kolmogorov-Smirnov test. All statistical analyses were undertaken with SPSS 20.0 software (IBM, Armonk, NY, USA), and a *p* < 0.05 was considered statistically significant.

## 3. Results

### 3.1 Clinical Features

**Fig. 1.**
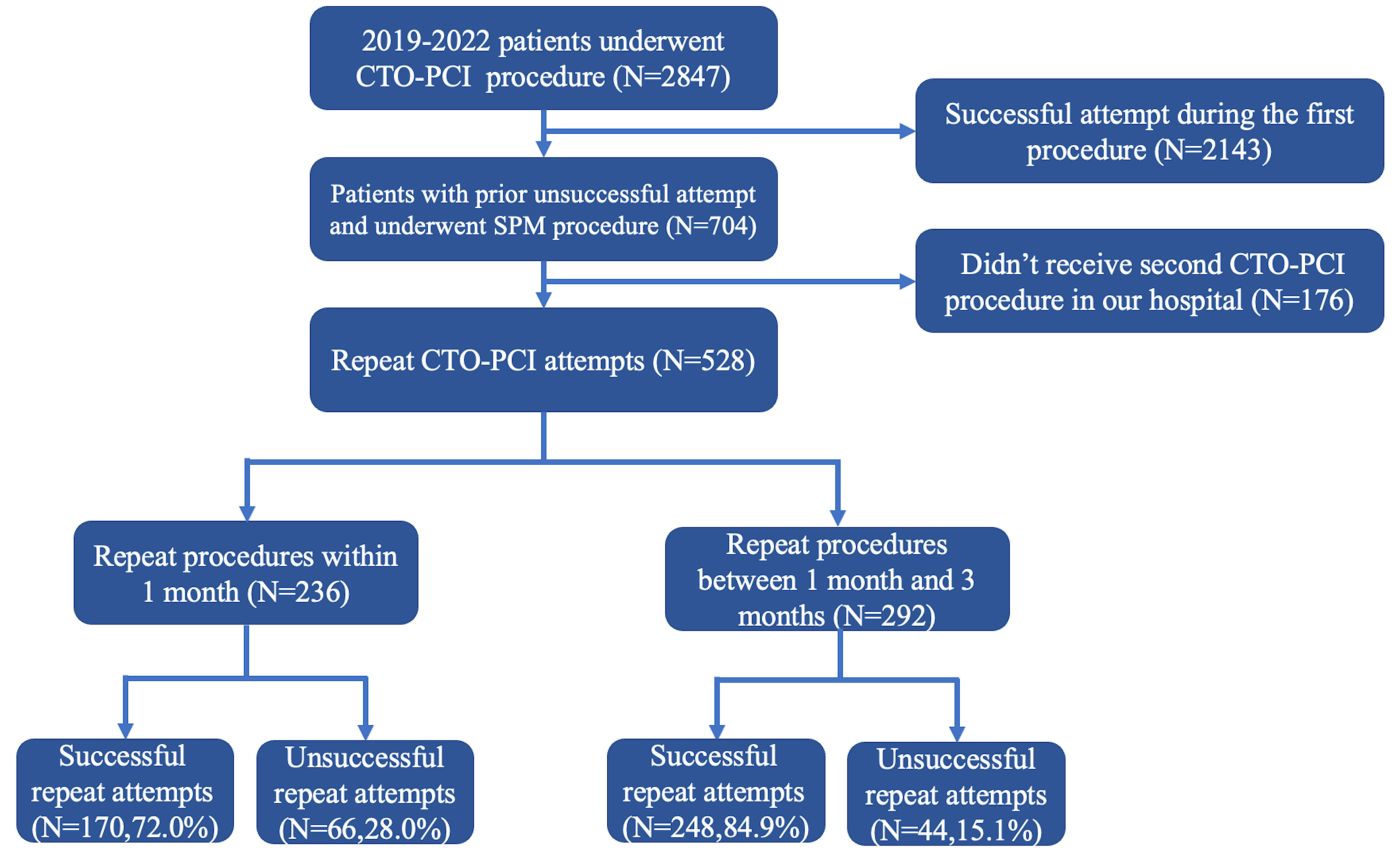
**Flowchart**. CTO-PCI, chronic total occlusion percutaneous coronary intervention; SPM, subintimal plaque modification.

We consecutively included 2847 patients who underwent CTO-PCI in our hospital from January 2019 to December 2022, among whom 704 patients underwent SPM due to unsuccessful initial procedures. Of these, 528 patients returned for a subsequent PCI at our hospital, with 236 undergoing the procedure within 1 month and 292 between 1 and 3 months. The flow chart illustrating these patient pathways is presented in Fig. 1. During the follow-up procedure, a total of 418 patients (79.2%) had their targeted lesion vessels successfully reopened. 89.6% of these individuals were male, with an average age of 61.0 ± 9.9 years. Additional baseline data, encompassing personal history, medical history, medication history, and certain laboratory test indicators, are detailed in Table 1.

**Table 1.** **Clinical characteristics of patients who underwent SPM procedures and received repeat coronary angiography/intervention**.

		Within 1 month (n = 236)	1 month–3 months (n = 292)	χ^2^	*p* value
Male, %		201 (85.2)	272 (93.2)	8.909	0.003
Age, %		62.3 ± 9.5	57.9 ± 12.7	-	0.007
Medical history, %					
	Hypertension	168 (71.2)	212 (72.6)	0.130	0.719
	Diabetes mellitus	53 (22.5)	70 (24.0)	0.168	0.682
	Dyslipidemia	174 (73.7)	234 (80.1)	3.052	0.081
	Prior MI	12 (5.1)	34 (11.6)	7.060	0.008
	Prior PCI or PTCA	75 (31.8)	89 (30.5)	0.103	0.748
	Prior CABG	16 (6.8)	12 (4.1)	1.853	0.173
Medication history, %					
	Aspirin, n (%)	236 (100)	292 (100)	-	-
	Clopidogrel or ticagrelor, n (%)	236 (100)	292 (100)	-	-
	Statin, n (%)	228 (96.6)	275 (94.2)	1.712	0.220
	ACEI/ARB, n (%)	102 (43.2)	116 (39.7)	0.657	0.417
	β‐Blockers, n (%)	75 (31.8)	72 (24.7)	3.295	0.069
	CCB, n (%)	33 (14.0)	46 (15.8)	0.322	0.571
	ARNI, n (%)	15 (6.4)	18 (6.2)	0.008	0.928
	Glucose-lowering drugs, n (%)	48 (20.3)	68 (23.3)	0.662	0.416
Personal history, %					
	Smoking, %	182 (77.1)	222 (76.0)	0.086	0.769
Laboratory examination					
	LDL-c, mmol/L	1.9 (1.5, 2.5)	2.0 (1.6, 2.4)	-	0.793

MI, myocardial infarction; PCI, percutaneous coronary intervention; PTCA, percutaneous coronary angioplasty; CABG, coronary artery bypass grafting; ACEI, angiotension converting enzyme inhibitors; ARB, angiotensin II receptor blockers; CCB, calcium channel blockers; ARNI, angiotensin receptor-neprilysin inhibitor; LDL-c, low-density lipoprotein cholesterol.

### 3.2 Angiographic and Procedural Characteristics in the Initial CTO-PCI Attempt

Table 2 summarizes the angiographic and procedural characteristics during the first CTO-PCI between the two groups. The culprit CTO lesions were mainly concentrated in the left anterior descending (LAD) and right coronary artery (RCA), which accounted for 44.5% and 50.6% respectively. During the initial attempt of the procedure, more than half of the patients received bilateral angiography, but there was no difference between the two groups (61.0% vs. 65.1%, *p* > 0.05). There was no significant difference in other characteristics including calcification, vessel tortuosity, occlusion length >20 mm, J-CTO score, knuckle wire, number of guide wires (GWs) used, SPM range, and SPM biggest Balloon size between the two groups. The median operation time of the patients who underwent the second operation within 1 month was 96 minutes, compared to 116 minutes in the other group, which was statistically significant (*p* < 0.05).

**Table 2.** **Initial angiographic and procedural characteristics**.

		Within 1 month (n = 236)	1 month–3 months (n = 292)	χ^2^	*t*	*p* value
CTO target vessels, %						
	LAD, n (%)	101 (42.8)	134 (45.9)	0.506	-	0.477
	LCX, n (%)	12 (5.1)	14 (4.8)	0.023	-	0.878
	RCA, n (%)	123 (52.1)	144 (49.3)	0.410	-	0.522
Procedural characteristics						
	Bilateral angiography, n (%)	144 (61.0)	190 (65.1)	0.922	-	0.337
	Calcification, n (%)	132 (55.9)	181 (62.0)	1.982	-	0.159
	Vessel tortuosity, n (%)	67 (28.4)	90 (30.8)	0.369	-	0.543
	Occlusion length >20 mm, n (%)	103 (43.6)	123 (42.1)	0.123	-	0.725
	J-CTO score	2.2 ± 0.6	2.3 ± 0.8	-	–1.137	0.256
	Knuckle wire, n (%)	17 (7.2)	25 (8.7)	0.329	-	0.566
	Number of GWs used, n	7.5 ± 0.8	7.6 ± 0.8	-	–1.697	0.090
	Attempted duration, min	96 (86, 103)	116 (104, 126)	-	-	<0.001
SPM characteristics & outcomes						
	SPM range (SPM length/lesion length)	0.6 (0.5, 0.8)	0.6 (0.6, 0.8)	-	-	0.297
	SPM biggest balloon size, mm	2.0 (1.5, 2.0)	1.5 (1.5, 2.0)	-	-	0.234

CTO, chronic total occlusion; LAD, left anterior descending; LCX, left circumflex artery; RCA, right coronary artery; GW, guide wires; J-CTO, J-chronic total occlusion score.

### 3.3 Technical Characteristics and Outcomes of Patients with Secondary CTO-PCI Procedure

The characteristics of the two groups of secondary PCI are shown in Table 3. The median time between operations was 22 days in the first month of the second operation and 69 days in the first three months. In addition, there were significant differences between the two groups in terms of referral to a high-volume operator (40.3% vs. 51.0%, *p* < 0.05), median attempted duration (*p* < 0.05), and median fluoroscopy time (*p* < 0.05) while there were no significant differences between the two groups in bilateral angiography and only an antegrade approach. In terms of repeat CTO results, there were differences in the successful revascularization (72.0% vs. 84.9%, *p* < 0.05) and perforation (8.9% vs. 6.2%, *p* = 0.232) while there was no difference between in-hospital MACE, death, acute myocardial infarction, stroke, repeat-PCI, emergency-coronary artery bypass grafting (CABG), pericardiocentesis, and the use of left ventricular (LV) assist devices (*p* > 0.05).

**Table 3.** **Technical characteristics and procedural outcomes of the repeat CTO-PCI**.

		Within 1 month (n = 236)	1 month–3 months (n = 292)	χ^2^	*p* value
Follow-up repeat CTO characteristics					
	Time of follow-up after SPM, days	22 (21, 27)	69 (59, 79)	-	<0.001
	Referral to high-volume operators, n (%)	95 (40.3)	149 (51.0)	6.090	0.014
	Bilateral angiography, n (%)	199 (84.3)	236 (80.8)	1.010	0.294
	Antegrade approach only, n (%)	108 (45.8)	126 (43.2)	0.360	0.548
	Attempted duration, min	110 (100, 115)	110 (104, 120)	-	0.039
	Fluoroscopy time, min	45 (35, 55)	55 (45, 57)	-	<0.001
Repeat CTO results, %					
	Successful revascularization, n (%)	170 (72.0)	248 (84.9)	13.164	<0.001
Complications, %					
	In-hospital MACE, n (%)	0	0	-	-
	Death, n (%)	0	0	-	-
	Acute myocardial infarction, n (%)	0	0	-	-
	Stroke, n (%)	0	0	-	-
	Repeat-PCI, n (%)	5 (2.1)	8 (2.7)	0.210	0.647
	Emergency-CABG, n (%)	0	0	-	-
	Perforation, n (%)	21 (8.9)	18 (6.2)	1.426	0.232
	Pericardiocentesis, n (%)	0	0	-	-
	LVAD use, n (%)	0	0	-	-
	None, n (%)	210 (89.0)	266 (91.1)	0.660	0.418

MACE, major adverse cardiovascular event; LVAD, left ventricular assist device.

**Fig. 2.**
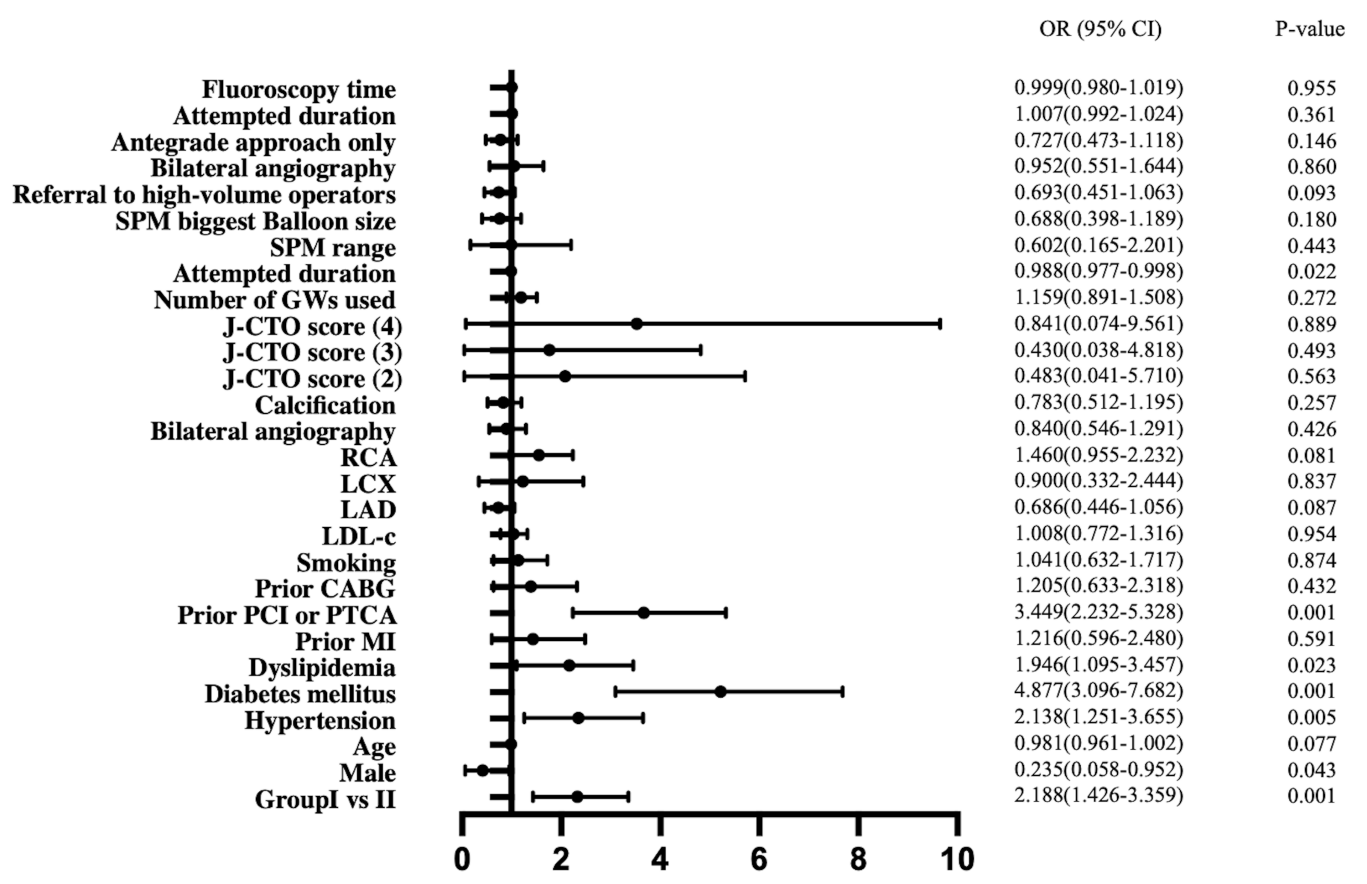
**Forest plot of variables associated with chronic total occlusion percutaneous coronary intervention (CTO-PCI) failure**. OR, odds ratio; CI, confidence interval.

We found a difference in the successful rate of revascularization between the two groups. After univariate logistic regression for the successful opening of occlusive lesions, we found that different groups, gender, hypertension, diabetes, dyslipidemia, prior PCI or PTCA, and duration of the first procedure were the factors that may have increased the success rate (Table 4 and Fig. 2). However, after adjusting for confounding factors, we found that groups, diabetes, dyslipidemia, and prior PCI or PTCA were the factors that affected the success rate of repeat CTO-PCI procedures (Table 4).

**Table 4.** **Univariate and multivariate logistic analysis**.

		Univariate		Multivariate	
		OR (95% CI)	*p-*value	OR (95% CI)	*p*-value
Group II vs. I		2.188 (1.426–3.359)	0.001	1.848 (1.023–3.477)	0.043
Male		0.235 (0.058–0.952)	0.043	0.605 (0.120–3.047)	0.543
Age		0.981 (0.961–1.002)	0.077		NS
Hypertension		2.138 (1.251–3.655)	0.005	1.607 (0.895–2.885)	0.112
Diabetes mellitus		4.877 (3.096–7.682)	0.001	4.493 (2.701–7.475)	0.001
Dyslipidemia		1.946 (1.095–3.457)	0.023	1.908 (1.007–3.616)	0.048
Prior MI		1.216 (0.596–2.480)	0.591		NS
Prior PCI or PTCA		3.449 (2.232–5.328)	0.001	2.570 (1.578–4.187)	0.001
Prior CABG		1.205 (0.633–2.318)	0.432		NS
Smoking		1.041 (0.632–1.717)	0.874		NS
LDL-c		1.008 (0.772–1.316)	0.954		NS
LAD		0.686 (0.446–1.056)	0.087		NS
LCX		0.900 (0.332–2.444)	0.837		NS
RCA		1.460 (0.955–2.232)	0.081		NS
Bilateral angiography		0.840 (0.546–1.291)	0.426		NS
Calcification		0.783 (0.512–1.195)	0.257		NS
J-CTO score					
	2	0.483 (0.041–5.710)	0.563		NS
	3	0.430 (0.038–4.818)	0.493		NS
	4	0.841 (0.074–9.561)	0.889		NS
Number of GWs used		1.159 (0.891–1.508)	0.272		NS
Attempted duration		0.988 (0.977–0.998)	0.022	0.998 (0.983–1.013)	0.767
SPM range		0.602 (0.165–2.201)	0.443		NS
SPM biggest Balloon size		0.688 (0.398–1.189)	0.180		NS
Referral to high-volume operators		0.693 (0.451–1.063)	0.093		NS
Bilateral angiography		0.952 (0.551–1.644)	0.860		NS
Antegrade approach only		0.727 (0.473–1.118)	0.146		NS
Attempted duration		1.007 (0.992–1.024)	0.361		NS
Fluoroscopy time		0.999 (0.980–1.019)	0.955		NS

## 4. Discussion

To the best of our knowledge, this study represents the first attempt to investigate the optimal timing interval for achieving favorable outcomes, including procedural success and adverse event rates, among CTO-PCI patients undergoing SPM for the first time. Our findings suggest that patients undergoing a second PCI shortly after SPM experienced lower intervention success rates and relatively higher complication rates. Conversely, adhering to guidelines recommending a procedure within 3 months resulted in the most satisfactory outcomes, particularly within a 1–3 months timeframe.

In recent years, significant advancements in wire technology, along with the utilization of dissection re-entry and retrograde approaches, have notably enhanced procedural success rates in CTO-PCI. Despite these advancements, a considerable number of patients still undergo CTO-PCI for the first time, facing challenges such as the inability of the guide wire to reach the true lumen during the procedure. In 2016, Wilson *et al*. [13] first introduced the concept of the “investment procedure”, defined as lesion modification of the proximal cap and/or CTO body through balloon angioplasty or the passage of a microcatheter. This intervention occurs before concluding the procedure in cases where CTO-PCI proves unsuccessful. Repeat angiography conducted after 1.5–4 months revealed the restoration of antegrade flow and healing of the dissection, which facilitated successful recanalization. This approach achieved a success rate of approximately 90% with acceptable rates of complications and MACE. Following this research, there was a gradual increase in studies investigating “investment” procedures. In 2015, Visconti *et al*. [14] introduced the term SPM, which became associated with these procedures, and subsequently resulted in better outcomes for this group of patients. In our study, SPM was employed in 42.8% of failed cases. Consistent with prior research, our study indicates that patients who underwent SPM experienced relatively fewer difficulties and higher success rates during subsequent attempts at recanalization. These favorable outcomes resulted in further research on SPM techniques. Xenogiannis *et al*. [15] observed that the likelihood of technical and procedural success in repeat CTO-PCI procedures conducted <60 days after SPM was lower (odds ratio (OR): 7.11; 95% confidence interval (CI): 1.36–37.16; *p* = 0.015). Conversely, they found that success rates were higher when repeat CTO-PCI attempts occurred ≥60 days after SPM. In our study, we identified a higher failure rate of second PCI surgeries within 30 days (HR: 1.848; 95% CI: 1.023–3.477; *p* = 0.043), aligning partially with the findings of Xenogiannis *et al*. [15]. Furthermore, the editorial comment in Hybrid 2.0 proposed that CTO-PCI can be re-attempted, typically after a 2–3 months interval following SPM [8], which corroborates with our findings.

A total of 528 patients with SPM were included in our study while Losif only included a relatively small sample size of 58. Instead of grouping the entire cohort, they initially conducted a descriptive study. Subsequently, when analyzed for technical and procedural success, they found that success rates were higher for procedures that took place ≥60 days after the index CTO-PCI (94% vs. 69%, *p* = 0.015). During univariable analysis, time to the subsequent procedure emerged as the sole factor linked to technical success. Notably, the focus was limited to procedural technique characteristics, and excluded the patients’ comorbidities and past medical history. This approach may have implications for the analysis of risk factors. In comparison, in our study, after multifactorial regression analysis, factors affecting the success of secondary CTO-PCI after SPM were also found to include hyperlipidemia [OR 1.908, 95% CI (1.007–3.616)], diabetes mellitus (DM) [OR 4.493, 95% CI (2.701–7.475)], as well as PCI or a history of PTCA [OR 2.570, 95% CI (1.578–4.187)]. All of these factors may affect the post-SPM CTO-PCI failure rate. Previous studies have demonstrated that in patients with comorbid diabetes mellitus, their endothelial cells are more susceptible to injury in the presence of high glucose due to impaired endothelium-dependent vasodilatation, increased inflammatory adhesion molecules, hyperosmolality, and oxidation of low-density lipoprotein (LDL) [16, 17, 18]. Diabetic patients are less likely to heal the damaged vascular endothelium after SPM, and therefore, patients with diabetes mellitus have a lower success rate during secondary CTO-PCI.

**Fig. 3.**
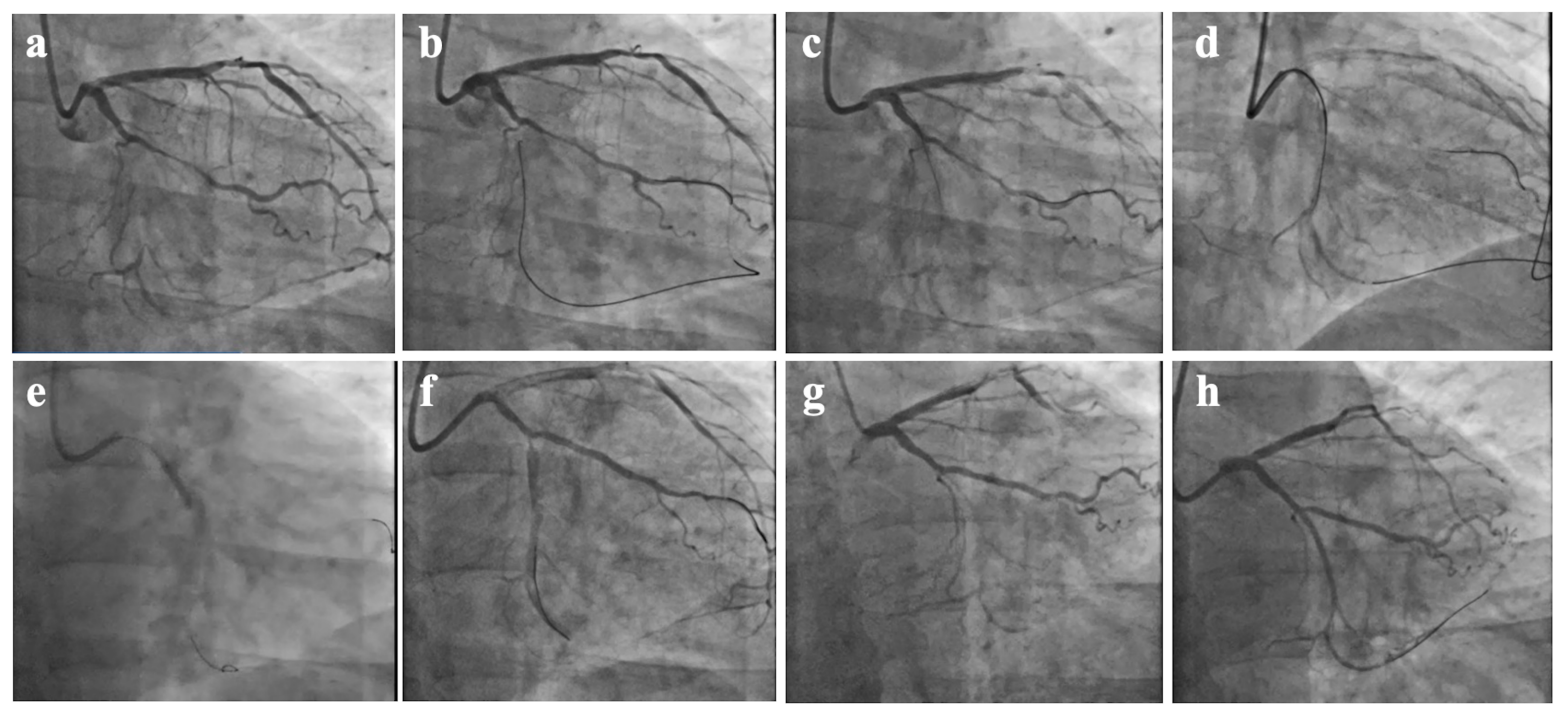
**CTO-PCI attempt, SPM procedure, and second operation at 65 days after SPM**. (a) Chronic total occlusion of the distal portion of the LCX. (b) The first attempt to open the LCX CTO through the occluded segment via the ipsilateral collateral circulation using the retrograde approach. (c) The retrograde guidewire is located under the endothelium. (d) Antegrade Dissection Re-entry technique into the true lumen. (e) SPM along the antegrade guide wire. (f) Postoperative SPM. (g) Second time of CAG after 65 days. (h) The LCX-CTO was successfully opened in the forward direction, and the re-operation was successful after SPM. CAG, coronary arteriography.

Zhong *et al*. [7] retrospectively analyzed 208 patients who underwent a failed CTO-PCI attempt and underwent a repeat procedure at the same cardiac center, among which 35 patients (16.8%) received SPM during the first attempt. They found that the interval between reattempts (increasing every 90 days) was inversely associated with the technical success rate of reattempts (OR: 0.85; 95% CI: 0.73–0.98; *p* = 0.030). Previous study [8] has shown that when the second attempt is over 90 days, the success rate decreased. This may be due to tissue proliferation and enhanced fibrosis during vessel healing, especially with medial or adventitial injuries [19]. Although 100% of patients were successfully opened within 60–90 days, only 2 of these patients underwent a second PCI. In 2020, Hirai *et al*. [20] conducted a study that focused on the impact of SPM in patients who underwent unsuccessful CTO-PCI. In their study, of all the patients who underwent the first CTO-PCI, 56 underwent a second angiography after the first PCI was unsuccessful. Of these 56 patients, a total of 31 (55.3%) underwent SPM. By comparison, only 44% of patients who underwent a failed CTO-PCI received SPM in our study. They found that the success rate of repeat CTO-PCI attempts was higher (87.1%) when the SPM procedure was performed at the index procedure. SPM was the only significant predictor of successful follow-up CTO-PCI attempts after an unsuccessful CTO-PCI attempt (Fig. 3). However, they did not specify the interval between the second surgery after SPM in their study. Our study found that attempts following 30–90 days after the initial procedure and SPM was a factor for the success of the second PCI attempt.

In terms of safety, our study found no difference in postoperative complications for immediate PCI or emergency CABG, as well as in the proportion of perforation in secondary operations after 30–90 days compared with patients receiving SPM within 30 days (8.9% vs. 6.2%, *p* = 0.232). A common failure mode of CTO-PCI is subintimal wire position with the inability to re-enter the true lumen distal to the CTO. Therefore, after attempting subintimal plaque modification via SPM, a second operation at an interval may improve the possibility of TIMI 3 flow. However, the main goal of CTO-PCI recanalization is to improve the patient’s ischemic symptoms [21]. It is also the common goal of operators and patients to avoid complications as much as possible. At an early stage during the procedure, after evaluating parameters including radiation dose, contrast volume, procedure time, and risk of the remaining treatment, operators may consider SPM as soon as possible [8]. Therefore, for patients willing to undergo SPM, a second attempt at CTO-PCI should be performed within the optimal time window. For those unable to undergo re-interventions within the appropriate timeframe, proactive management of other risk factors should be considered, including intensive lipid-lowering therapy and glycemic control for patients with diabetes. By adopting these measures, more optimal outcomes can be achieved for CTO patients.

In our study, the median operation time of the two groups was 96 minutes and 116 minutes, which were less than the 3–4 hours recommended in hybrid 2.0, which may be related to the operation time predicted by the surgeons. The CTO operators in our center may depend on the estimated operation time and other factors after trying various schemes. The operation time of the first CTO-PCI was shortened as much as possible and the SPM method expeditiously performed, hoping to minimize the amount of radiation for patients and operators reduce the incidence of complications. The risk in the SPM process is the occurrence of perforations, both at the proximal and distal end of the occlusion. CTO-PCI carries an increased risk of complications in comparison with non-CTO-PCI, especially perforation [22]. Across multiple contemporary registries, the incidence of cardiac tamponade in different studies is not consistent, which is closely related to the difficulty of the lesion [14, 23, 24]. In previous study, when analyzing the success rate and safety of the SPM operation, pericardial effusion is one of the most common complications, as noted by Xenogiannis *et al*. [15]. In their study, postoperative pericardial effusion accounted for 1.7%, while in-hospital MACE events were the most important, accounting for 3.3%. In addition, Hirai *et al*. [25] noted in their article that the incidence of MACE is 6.8%, and perforation accounts for 4.7%. The incidence of perforation in our study is higher than that in the above study. This may be related to the fact that our patients had more calcification and more complex lesions. In terms of safety, enhancing bilateral imaging, using a knuckled (J-shaped) guidewire, changing to the retrograde approach [26], and performing the CTO-PCI by a skilled physician and team [27] may result in fewer adverse events.

## 5. Limitation

This study has several limitations. First, our study was a retrospective cohort study that included a relatively small number of patients. Second, the outcome in this study was limited to the success and safety of the CTO operation while improvement of symptoms was not reported. Finally, our study was conducted at only one of our hospitals, and it is possible that in the future, as the number of operators treating CTOs increases, data from multiple centers could be included so that more accurate conclusions can be obtained.

## 6. Conclusions

We found that approximately one-fourth of CTO patients failed to achieve successful vessel recanalization during the initial attempt. Among those who underwent SPM following the initial unsuccessful attempt, we observed that patients who returned for a second CTO-PCI within 1 month had lower procedural success rates compared to those who underwent the procedure within 1–3 months while there were no significant differences in complications including MACE, pericardial effusion between those two groups. Additionally, diabetes, hyperlipidemia, and a history of previous PCI or PTCA were identified as risk factors for procedural failure among CTO-PCI patients who received SPM.

## Availability of Data and Materials

The datasets used and/or analyzed during the current study are available from the corresponding author on reasonable request.
